# MyNutriCapsule: An innovative approach to reduce water and nutrient waste in fertigation farming

**DOI:** 10.1016/j.ohx.2025.e00711

**Published:** 2025-09-28

**Authors:** Azlan Abdul Aziz, Nor Azriani Mohamad Nor, Wan Nurshazelin Wan Shahidan, Siti Nor Nadrah Muhamad, Nur Syuhada Muhammat Pazil, Syazwani Ya, Nur Faezah Omar

**Affiliations:** a Faculty of Computer and Mathematical Sciences, Universiti Teknologi MARA (UiTM) Cawangan Perlis, Arau 02600 Perlis, Malaysia; b Faculty of Computer and Mathematical Sciences, Universiti Teknologi MARA (UiTM) Cawangan Melaka Kampus Jasin, Merlimau 77300 Melaka, Malaysia; c Faculty of Business and Management, Universiti Teknologi MARA (UiTM) Cawangan Perlis, Arau 02600 Perlis, Malaysia; d Faculty of Plantation and Agrotechnology, Universiti Teknologi MARA (UiTM) Cawangan Perlis, Arau 02600 Perlis, Malaysia; e Statistical Analytics, Forecasting and Innovation (SAFI) Research Interest Group, Universiti Teknologi MARA Cawangan Perlis, Arau 02600 Perlis, Malaysia

**Keywords:** Low cost, MyNutriCapsule, Nutrient optimisation, Precision farming, Wastage

## Abstract

Although fertigation offers substantial efficiency and productivity benefits, it also has significant drawbacks. Setting up a fertigation system requires considerable costs and knowledge and professionalism in managing soil, water, and fertiliser. Inefficient fertigation practices can also lead to groundwater contamination from nutrients leaching beyond the root zone. Consequently, this study aimed to introduce the MyNutriCapsule, an innovative product designed by Mr. Azlan Abdul Aziz, a senior lecturer from Universiti Teknologi MARA, Malaysia. The product was primarily employed to significantly reduce water and nutrient waste in fertigation agriculture. MyNutriCapsule recorded 98% and 18% reduction in fertiliser and liquid nutritional fertiliser wastage, respectively. The product also documented a 20% reduction in treated water consumption and a 27% decrement in overall operational costs. The observations validated the efficiency of MyNutriCapsule in promoting sustainable and cost-effective agricultural practices.

## Graphical abstract

**Figure:**
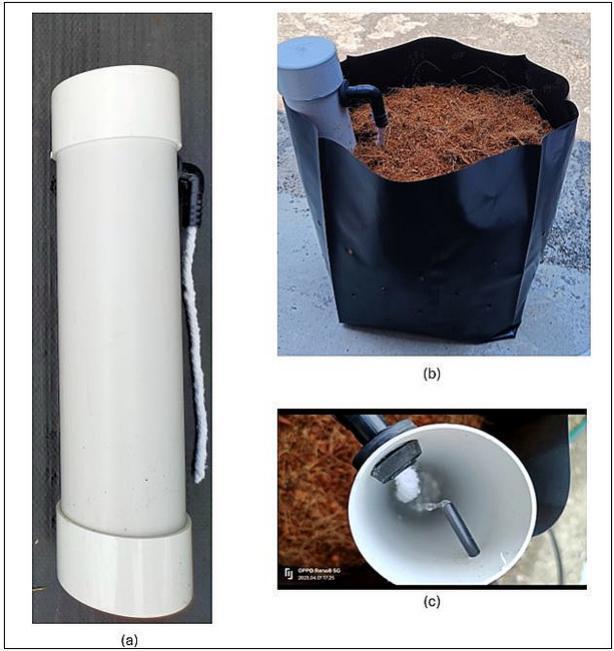

Specifications table

**Table:** 

**Specifications Table Hardware name**	MyNutriCapsule
**Subject area**	Environmental, planetary, and agricultural sciences
**Hardware type**	Nutrient container
**Closest commercial analogue**	No commercial analogue is available
**Open-source license**	GNU General Public License (GPL)
**Cost of hardware**	MYR 8.55 (∼2.00 USD)
**Source file repository**	https://data.mendeley.com/datasets/m4zthdz8tf/1

## 1 Hardware in context

The word fertigation is the combination of “fertiliser” and “irrigation”, which is a technique of applying fertiliser through an irrigation system [1]. A notable efficiency was observed when employed in drip and sprinkler irrigation systems, ensuring uniform nutrient distribution and effective water and fertiliser utilisation [[2], [3], [4]]. The fertigation concept dates back to Roman times, when urban sewage was employed to irrigate crops, providing water and nutrients [5]. Subsequently, farmers in the Jordan Valley in the early 1930 s placed jute bags filled with ammonium sulphate at the entrance of irrigation canals to fertilise their banana plants. Over the past 15 years, the irrigation technique has evolved substantially, particularly in water-scarce areas. The need for efficient water and fertiliser usage has contributed to the development, leading to improved crop yields and diminished labour costs [4,[6], [7], [8]].

Various fertigation devices, including Venturi injectors, differential pressure tanks, and proportional fertilisation pumps, have been developed to elevate the efficiency and accuracy of nutrient application [8]. Ongoing research also aims to enhance fertigation techniques, including integrating modern technologies such as the Internet of Things (IoT) to produce smart fertigation systems [[9], [10], [11]]. The fertigation approaches in Malaysia exhibit substantial efficiency and productivity benefits. The fertigation method is particularly significant in Malaysia, as it can enhance crop yields and resource management and facilitate sustainable agricultural practices. Integrating technologies, such as IoT and smart systems, can also further optimise the fertigation process, offering a promising solution for small-scale farmers and large agricultural enterprises.

Employing IoT in fertigation systems allows real-time monitoring and management of nutrient concentrations and pH levels, significantly improving fertiliser utilisation efficiency. The technology also reduces labour and time. Furthermore, optimal plant nutrient levels are ensured through the implementation of the advancement, as demonstrated by a Malaysian IoT-based system that applied NodeMCU ESP32 microcontrollers [12].

Smart fertigation systems, including fuzzy logic for irrigation decisions, reportedly enhanced irrigation efficiency and crop yields. For instance, a smart chilli cultivation system in urban greenhouses documented improved irrigation management and chilli production [13]. Systems that automate fertiliser mixing with Arduino and Proportional Integral Derivative (PID) controllers also aid in maintaining desired salinity and pH levels, further elevating the accuracy of fertigation practices [14].

Although the initial cost of installing fertigation systems is substantial, it offers increased net incomes due to increased crop yields. For example, growing chillies with fertigation under rain shelters and open systems recorded superior net income to conventional methods [15]. Fertigation systems are also economically viable for smallholders. Among farmers in Kelantan, 70 % indicated willingness to adopt the technology [16].

Although fertigation offers numerous advantages, the technique has significant disadvantages. For instance, effective fertigation requires exceptional knowledge and professionalism in managing soil, water, and fertiliser. The complexity can be a barrier for farmers and technicians with inadequate expertise required [17,18]. The initial cost of setting up a fertigation system can also be significant. A fertigation system requires injectors, such as fertiliser tanks, fertigation pumps, and venturi injectors, which vary in cost and efficiency [7,19].

Over-fertilisation in fertigation can lead to nutrient runoff and environmental pollution, particularly for nitrogen and phosphorus fertilisers, which can result in considerable ecological damage [[20], [21], [22]]. Inefficient fertigation practices can also lead to nutrient leaching beyond the root zone, contaminating groundwater. The phenomenon is especially concerning heavy rainfalls, when the possibility of leaching increases [1,23]. Consequently, the predominant objective of this study is to introduce and evaluate the effectiveness of MyNutriCapsule, an innovative product developed by Mr. Azlan Abdul Aziz, a senior lecturer from Universiti Teknologi MARA, Malaysia. The primary function of the product is to considerably reduce water and nutrient waste in fertigation agriculture.

## 2 Description of hardware

**Fig. 1:**
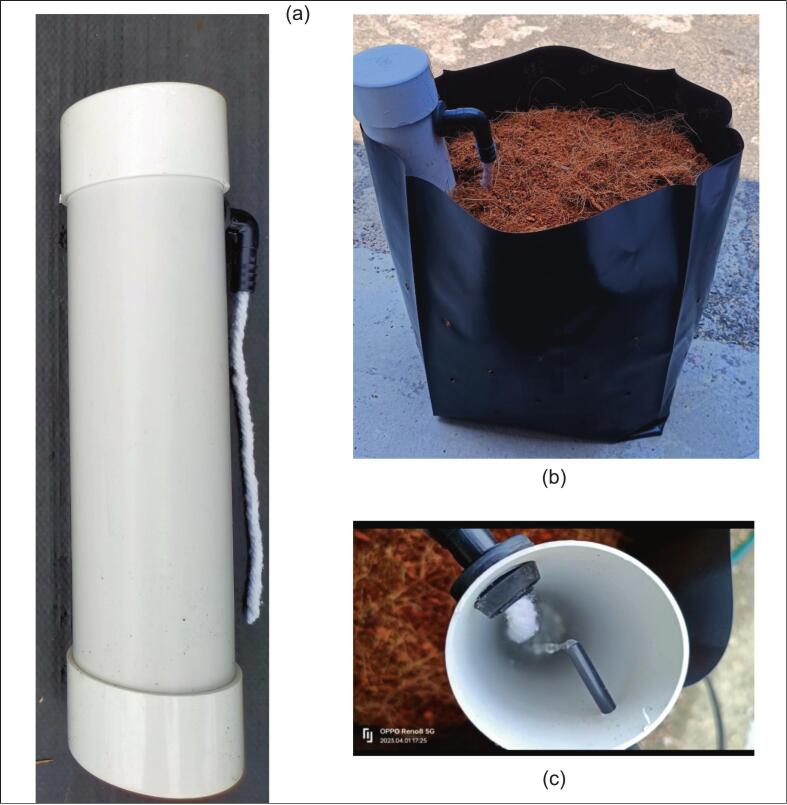
(a) An overview of MyNuriCapsule, (b) the MyNutriCapsule in a polybag, and (c) the flow of water or liquid nutrient fertiliser into MyNutriCapsule.

Fig. 1 illustrates MyNutriCapsule, a novel, low-cost, innovative fertigation device designed to optimise nutrient delivery in fertigation farming systems. The system was officially registered under the Intellectual Property Corporation of Malaysia (MyIPO) (Copyright No: CRAR00004864). Functioning as a storage unit for liquid nutritional fertilisers, MyNutriCapsule delivers nutrients directly to the plant root zone through a controlled absorption mechanism.

MyNutriCapsule is constructed with readily available and affordable components, including a 50 mm (diameter) × 16-inch (406.4 mm) unplasticised polyvinyl chloride (uPVC) pipe, two 50 mm uPVC end caps, a 16 mm elbow joint, and a rubber grommet. A cotton string is also employed, which is typically repurposed from floor mops. The uPVC pipe length is adjustable based on the polybag size. A standard 16-inch pipe unit is capable of storing between 500 mL and 1000 mL of liquid fertiliser.

**Fig. 2:**
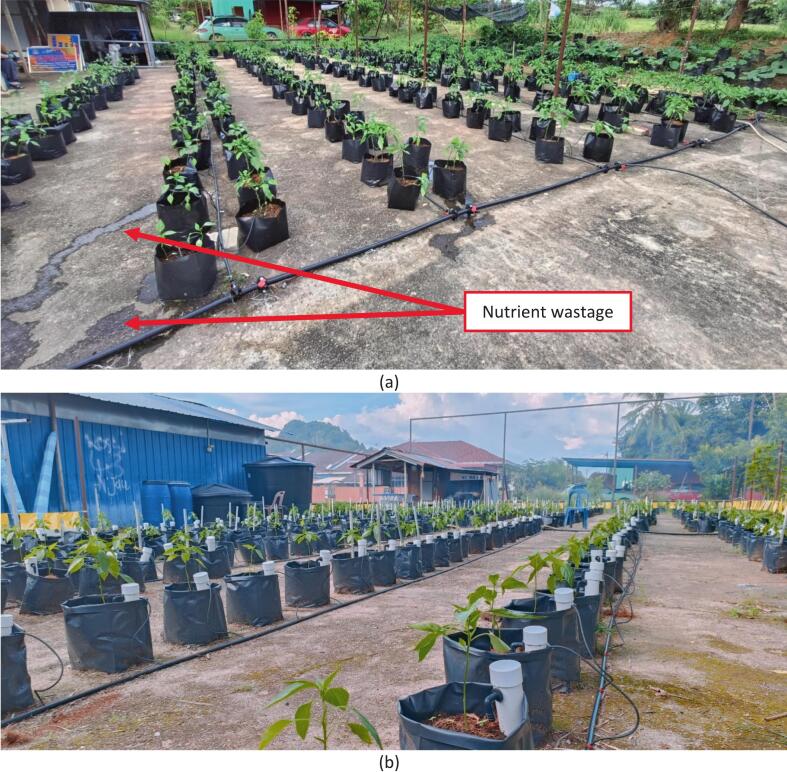
(a) Leakages due to rapid nutrient flow and (b) no nutrient leaks with MyNutriCapsule installation.
(iii) During rainy seasons, rainwater dilutes the fertiliser concentration in the polybags, prompting farmers to increase fertiliser strength. The step increases the costs and risks of nutrient imbalance, affecting plant health.

The development of MyNutriCapsule was in direct response to several inefficiencies in conventional fertigation systems, which commonly rely upon drippers for delivering nutrients. The traditional systems exhibit the following limitations:

(i) Nutrient concentration is frequently uneven, with excessive accumulation at the point where the dripper penetrates the planting media, leading to poor nutrient distribution across the polybag.
(ii) Rapid nutrient flow commonly results in leakages in the polybags, resulting in significant wastage [see Fig. 2(a)].

Among the critical improvements offered by MyNutriCapsule are as follows:

(i) Nutrient absorption regulation is achieved with a cotton string that directly delivers fertiliser to the root zone. The system only activates when the planting medium is dry.
(ii) Minimisation of leaching and over-acidification of the planting medium risks [see Fig. 2(b)].
(iii) Ensures consistent nutrient concentration even during rainy seasons, an advantage over conventional approaches that necessitate frequent fertiliser concentration adjustments due to dilution from rainwater.

Although the initial setup cost can be slightly higher than traditional methods, MyNutriCapsule offers notable long-term savings from fertiliser usage and wastage reductions. The device is also durable and requires low maintenance. The cotton string in the MyNutriCapsule device only necessitates periodic replacement, making it cost-effective for up to four to five years of continuous employment. Furthermore, MyNutriCapsule is superior to existing fertigation systems, considering its simplicity, precision in nutrient delivery, sustainability, and adaptability to smallholder farming conditions. The customisable design of the product also allows further innovation in controlled-release irrigation technologies and accurate agriculture solutions.

In summary, MyNutriCapsule provides valuable benefits to farmers, researchers, and practitioners, particularly in the field of agriculture. The following key points highlight its practical applications:

• MyNutriCapsule significantly reduces nutrient and water wastage in fertigation-based farming systems.
• It helps lower overall operational costs compared to conventional fertigation methods.
• The growing medium (cocopeat) maintained a stable, neutral pH level when integrated with MyNutriCapsule.
• Plants grown with MyNutriCapsule showed improved growth performance, with visibly healthier foliage and higher crop yields compared to those without the system.

## 3 Design files summary

No custom or three-dimensional (3D)-printed components are required for constructing MyNutriCapsule. The device utilises standard, off-the-shelf parts that can be bought from local hardware and plumbing supply stores or widely available through online marketplaces. Consequently, no design files are necessary, contributing to the notable accessibility and cost-effectiveness of the product for broad implementation and replication.

## 4 Summary of material costs

Table 1 summarises the material costs required to set up MyNutriCapsule. Most of the components are readily obtainable from local hardware and plumbing supply stores, ensuring the device remains highly accessible and cost-effective.

**Table 1:** Summary of Material Costs for MyNutriCapsule.

**Material**	**Component**	**Number**	**Cost per unit**	**Total cost**	**Source of material**	**Material type**
uPVC pipe Diameter: 50 mm Length: 16 in	−	1	MYR 2.00	MYR 2.00	Local hardware and plumbing supply stores	Polymer (uPVC)
uPVC end caps	−	2	MYR 2.50	MYR 5.00	Local hardware and plumbing supply stores	Polymer (uPVC)
Fertigation fitting elbow joint	−	1	MYR 1.00	MYR 1.00	Online stores	Polymer
Rubber grommet	−	1	MYR 0.50	MYR 0.50	Online stores	Polymer
Cotton string	−	1	MYR 0.05	MYR 0.05	Local hardware and plumbing supply stores	Cotton
**Total**				**MYR 8.55**		

### 4.1 Assembly instructions

Typically, an individual can complete the straightforward construction of MyNutriCapsule within approximately 20 min. The following is the step-by-step assembly procedure:

**Table:** 

Step 1:	Securely attach one uPVC end cap unit to the bottom end of the uPVC pipe with an appropriate adhesive. Ensure the bond is completely sealed without gaps to prevent water leakage from the capsule.
Step 2:	Measure and mark a point 4 cm from the top of the uPVC pipe before drilling a 16 mm diameter hole. Install a rubber grommet securely into the hole and insert an elbow joint. Thread a piece of cotton string through the elbow joint. The string should extend to and be in contact with the bottom of the inside of the capsule. The string facilitates absorption of nutrient solution through capillary action.
Step 3:	Directly opposite the previously drilled hole in Step 2, drill another hole of a 4 mm diameter.
Step 4:	Securely fit a second uPVC end cap to cover the top opening of the MyNutriCapsule. This is to ensure protection against rainwater entry. The step also hinders pests from making the capsule a breeding ground for pests, particularly Aedes mosquitoes.

### 4.2 Safety considerations

Employ personal protective equipment (PPE), such as safety glasses and gloves, during drilling and glueing operations to avoid injury. Avoid inhalation of harmful fumes by ensuring adequate ventilation when applying adhesives. Refer to the components detailed in the design files summary and the summary of material costs provided to ensure accurate replication of the MyNutriCapsule.

## 5 Operation instructions

Upon completing the assembly of the MyNutriCapsule, the following guidelines are provided for the safe and effective utilisation of the system:

**Table:** 

Step 1:	Place the completed MyNutriCapsule securely into a polybag containing cocopeat, which is a coconut husk-based growing medium that is rich in fibre and has excellent water retention capabilities, ideal for holding liquid nutrient fertilisers.
Step 2:	Insert the pre-assembled 4 mm microtube connected to the fertigation polypipe system into the 4 mm hole drilled in Step 3 of the building instructions.
Step 3:	Following the appropriate arrangement of all polybags containing MyNutriCapsules, activate the water pump to facilitate the flow of water or liquid nutrient fertiliser through the system.
Step 4:	During the initial utilisation, carefully determine and record the duration required for the nutrient solution to fill the MyNutriCapsule. The documented time will facilitate future irrigation cycle optimisations.
Step 5:	Adjust irrigation frequency according to plant age and weather conditions. The frequency of irrigation should typically be increased proportionally as the plants mature.

### 5.1 Safety considerations

Ensure the water pump and electrical connections are dry and isolated to prevent electrical hazards. Inspect the system for leaks or blockages regularly and promptly address any issues to maintain optimal functionality.

## 6 Validation and characterisation

The effectiveness of MyNutriCapsule has been in chilli fertigation farming in a community project at Kawasan Rukun Tetangga (KRT) Taman Bukit Kayangan, Perlis, Malaysia. The innovative product has enhanced plant growth by delivering a consistent and direct supply of liquid nutrient fertiliser to the root zones of 500 polybags planted with *Capsicum Annum L.* Var. Kulai. Furthermore, the MyNutriCapsule implementation has significantly elevated nutrient usage efficiency and water conservation compared to conventional fertigation techniques. Various institutions have also recognised the effectiveness and positive effects of MyNutriCapsule utilisation. Notable achievements recorded by the product are detailed as follows:

(i) Awarded 3rd place at the Anugerah Perdana Rukun Tetangga in the Best Innovation Project category, which was organised by the Ministry of National Unity, Malaysia.
(ii) Received a gold medal at the 13th International Innovation, Invention and Design Competition 2024.

The primary objective of MyNutriCapsule production is wastage minimisation and enhancing the liquid nutritional fertiliser employment efficiency in fertigation farming. This study manually recorded data on selected variables (3rd – 8th November 2024) to evaluate the effectiveness of MyNutriCapsule. The following are the variables observed:

(i) The nutrient wastage levels in the plots with MyNutriCapsule (Plot A) and conventional fertigation methods (Plot B)
(ii) The volume of liquid nutrient concentrate utilised in both plots
(iii) The treated water levels required to dilute the nutrient solution
(iv) The total costs of setup and operations associated with each plot

In this study, 100 polybags each were employed in Plots A and B. Plot A had MyNutriCapsule installed, while Plot B was without MyNutriCapsule. Plot B employed a conventional fertigation system commonly used in Malaysia. The irrigation setup utilised a 1 horsepower (hp) water pump with 1-inch inlet and outlet fittings, and the irrigation was scheduled twice daily, with each session lasting approximately 5 min. To accurately measure excess water, a collection basin was placed beneath each polybag in both Plot A and Plot B. This allowed for systematic recording of nutrient and water wastage.

Observations and recorded data revealed that polybags in Plot B experienced an average nutrient wastage of 125 L per week, whereas Plot A, which employed the MyNutriCapsule, recorded only 2.5 L. This represents a substantial reduction of 122.5 L, equivalent to 98 % (Table 2). These findings clearly demonstrate the effectiveness and capability of MyNutriCapsule in significantly minimising nutrient wastage.

**Table 2:** Comparative Analysis of Nutrient Solution and Resource Utilization Between Plot A (MyNutriCapsule) and Plot B (Conventional System).

**Variable**	**Plot A (with MyNutriCapsule)**	**Plot B (without MyNutriCapsule)**	**Difference**
Nutrient wastage (L/week/polybag)	2.5	125	98 % wastage reduction
Nutrient concentrated used (L/week)	36	45	20 % reduction
Treated water utilised for nutrient dilution (L/week)	800	1000	20 % reduction
Total operational cost (nutrient concentrated, and treated water, MYR/month)	75	95	21.05 % saving

In terms of nutrient preparation, the standard irrigation system initially required 1,000 L of treated water mixed with 45 L of nutrient concentrate, maintaining a ratio of 1 L of treated water to 0.045 L of concentrate. Both Plot A and Plot B began with identical 1,000 L tank capacities. However, after one week, the tank in Plot B was fully depleted, whereas Plot A still had 200 L remaining. This indicates that Plot A consumed only 800 L of nutrient solution and 36 L of nutrient concentrate—demonstrating a 20 % reduction in usage compared to Plot B (Table 2). Overall, MyNutriCapsule offers a reduced overall operational cost of up to 27 % or MYR 20 per month.

**Fig. 3:**
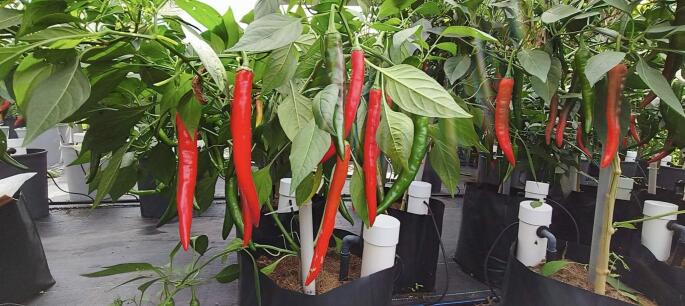
The quality of *Capsicum annuum L.* Var. Kulai after MyNutriCapsule is applied.

Qualitative observations from field applications support the quantitative performance improvements provided by MyNutriCapsule. Based on the results recorded in this study, the growing medium (cocopeat) with MyNutriCapsule installed remained within a neutral pH range. Consequently, excessive acidity was prevented, which could affect root health. Furthermore, the plants from Plot A exhibited enhanced vigour, demonstrating visibly healthier foliage and increased crop yields than the plants from Plot B (see Fig. 3).

Although boasting numerous advantages, MyNutriCapsule also has limitations. A concern is the growth of algae on the cotton string, particularly when exposed to direct sunlight. Over time, the algae can hinder nutrient absorption efficiency. A practical solution to the matter involves connecting a 16 mm polypipe to the elbow joint, shielding the cotton string from light exposure and preventing algae proliferation.

Currently, MyNutriCapsule has only been implemented for growing *Capsicum Annum L. Var. Kulai*, a crop that is in significant demand and price in Malaysia. Consequently, the nutrients and costs discussed in this study specifically refer to the crop and may vary when implemented on other plants.

Nevertheless, MyNutriCapsule can still offer substantial nutrient and cost savings when adapted for other crops, such as eggplant and okra. MyNutriCapsule is also suitable for implementation in tropical climate countries, including Malaysia, Indonesia, Singapore, Thailand, Myanmar, and the Philippines. Nonetheless, future studies can consider employing high-water-requirement crops, particularly in high temperatures, without additional water requirements.

## Ethics statements

No ethical approval is needed.

## CRediT authorship contribution statement

**Azlan Abdul Aziz:** Writing – original draft, Methodology, Formal analysis, Data curation, Conceptualization. **Nor Azriani Mohamad Nor:** Writing – review & editing. **Wan Nurshazelin Wan Shahidan:** Writing – review & editing. **Siti Nor Nadrah Muhamad:** Writing – review & editing, Formal analysis. **Nur Syuhada Muhammat Pazil:** Writing – review & editing, Formal analysis. **Syazwani Ya:** Writing – review & editing. **Nur Faezah Omar:** Writing – review & editing.

## Declaration of competing interest

The authors declare that they have no known competing financial interests or personal relationships that could have appeared to influence the work reported in this paper.
